# Assessment of CAD/CAM Fabrication Technologies for Post and Core Restorations—A Narrative Review

**DOI:** 10.3390/medicina60050748

**Published:** 2024-04-30

**Authors:** Mariya Dimitrova, Angelina Vlahova, Rada Kazakova

**Affiliations:** 1 Department of Prosthetic Dentistry, Faculty of Dental Medicine, Medical University of Plovdiv, 4000 Plovdiv, Bulgaria; angelina.vlahova@mu-plovdiv.bg (A.V.); rada.kazakova@mu-plovdiv.bg (R.K.); 2 CAD/CAM Center of Dental Medicine, Research Institute, Medical University of Plovdiv, 4000 Plovdiv, Bulgaria

**Keywords:** post and core restorations, CAD/CAM, semi-digital, prosthodontics, digital dentistry

## Abstract

The primary objective of this study is to conduct a comprehensive review of the existing literature that discusses research on post and core restorations, covering aspects such as their composition, manufacturing methods, and clinical effectiveness. The methodology employed in this review encompasses the implementation of a well-defined search strategy, the establishment of criteria for inclusion and exclusion, and the selection of relevant studies to summarize their findings. To gather relevant literature published between 1993 and 2023, the research team conducted separate searches on PubMed, Web of Science, and Embase databases. In total, 168 titles were initially retrieved from these electronic databases. By applying the predefined exclusion criteria, the researchers identified 73 articles that specifically address the conventional and computer-aided design/computer-aided manufacturing (CAD/CAM) technologies employed in post and core restorations. These treatments are commonly employed to restore teeth that have received endodontic therapy and subsequently experienced loss of dental structure. The development of computerized technology for the creation of customized posts and cores has emerged as a straightforward and efficient alternative to traditional methods. The review synthesizes papers discussing the techniques and materials involved in CAD/CAM-based construction of post and cores. It explores strategies for restoring endodontically treated teeth, highlighting both direct and indirect approaches. Commonly mentioned materials include zirconia, composite resin, and hybrid ceramics. Despite the limited literature on CAD/CAM post and core procedures, the review emphasizes the necessity of further research to assess long-term outcomes and efficacy. Additionally, it suggests including implications for future research and clinical recommendations to enhance the depth and practical relevance of the review.

## 1. Introduction

The review explores fabrication technologies for post and core restorations, particularly focusing on customized cast post and cores [1]. These restorative procedures are commonly employed to manage significant tooth damage resulting from conditions like untreated tooth decay, dental trauma, poor oral hygiene, acidic foods and drinks, and certain medical conditions like acid reflux or eating disorders [2]. The rationale behind their widespread use lies in their ability to provide individualized solutions for cases involving elliptical or flared canals, where standard prefabricated posts may not offer optimal adaptation. Additionally, custom-cast post and core configurations offer heightened adjustability, leading to enhanced resistance against torsional stresses. This adjustability is especially beneficial for stabilizing structures between the crown and root, particularly in single-rooted and premolar teeth where reductions in tooth structure during preparation can weaken the tooth [3,4]. Furthermore, tailored post and core restorations demonstrate significant resistance against rotational stresses in multi-rooted teeth with substantial loss of tooth structure [5,6]. This context underscores the importance of exploring and understanding the fabrication techniques associated with custom-cast post and core restorations to address specific clinical challenges effectively [7].

An ideal post and core should exhibit attributes such as enhanced crown retention, biocompatibility, non-toxicity, and superior tensile strength, alongside resistance to the fatigue induced by occlusal and shear loads [8]. Additionally, a well-designed post should evenly distribute stresses across the adjacent root surface and extend apically to at least the height of the crown or two-thirds the length of the root [9,10]. This distribution of stress serves to manage occlusal loads and bolster resistance. Moreover, the color of the post and core should ideally match that of natural dentin, a particularly significant consideration for anterior tooth restorations [11,12]. 

The production of customized post and cores can be accomplished through either a direct approach utilizing a resin model or an indirect technique employing elastomeric impressions of the prepared canal [13]. This impression is subsequently replicated in gypsum by a laboratory technician, who crafts a wax pattern that is then cast into a metal alloy to create the post and core. On the other hand, the direct method entails the clinician using auto-polymerizing acrylic resin to shape an acrylic pattern [3]. This pattern is then dispatched to the laboratory for the stages of burnout and casting. The direct technique offers enhanced anticipatory capabilities for the clinician, as the cast post-and-core pattern is physically assessed and confirmed for its fit on the actual tooth before the casting procedure takes place [14]. The key advantage of using customized post and cores lies in their ability to fit a wide range of teeth, including those with oval canals, and their ease of removal during potential retreatment [15]. Furthermore, both the post and core functions are in unison, diminishing the likelihood of core separation [16]. Even when dealing with proclined teeth, the angulation of the core within cast post and cores can be adjusted to match the crown’s contour [17].

A study conducted by Balkenhol et al. revealed that teeth restored with custom-cast post and cores exhibited a promising long-term prognosis, boasting a survival rate of 7.3 years [18]. Similarly, Dietschi et al. [19] and Maccari et al. [20] showcased the high fracture resistance of teeth restored with custom-cast post and cores in similar experimental settings. However, it is important to note that cast metal posts demand additional chairside and laboratory time, contributing to higher treatment costs [21,22]. Customized cast post and core restorations offer a good solution for addressing significant tooth damage, showcasing a blend of historical significance and modern technological advancements that contribute to their clinical success and positive long-term outcomes.

Throughout more than two and a half centuries, historical accounts bear witness to the intriguing evolution of embedding posts within fractured tooth roots. This practice can be traced back to the ingenious mind of Pierre Fauchard, a luminary in modern dentistry, who first introduced this concept in the early months of 1728 [23]. Building upon this foundation, French dentist Claude Mounton, in 1745, contributed to the narrative by detailing the idea of affixing a gold crown to a gold post implanted within the root structure [24]. As time progressed into the 19th century, there emerged a shift from the use of metal poles to wooden posts, a development driven by both innovation and necessity. However, this transition encountered a significant setback owing to wood’s susceptibility to moisture absorption, a characteristic that inevitably led to expansion and the perilous occurrence of root fractures [25].

Fast-forwarding through the pages of dental history, we encounter the contributions of C.M. Richmond, an American dental visionary. Richmond’s legacy was marked by introducing what is now known as the “Richmond crown”. This innovative creation embodied a single-piece, post-retained crown that not only displayed a porcelain facing but also fulfilled the role of a bridge retainer, underscoring the convergence of practicality and artistry in dental restoration [26]. A monumental leap in the realm of dental innovation occurred in the 1930s, a period marked by the emergence of custom-cast posts and cores as a groundbreaking development [27]. This novel approach entailed the fabrication of the post and core as distinct entities, effectively diverging from the traditional amalgamation. This departure from convention yielded a marked improvement in the marginal adaptation of the final restoration, ushering in a new era of precision and efficacy in dental restorative techniques [28]. This historical trajectory underscores not only the evolution of materials and techniques but also the enduring quest for refining dental treatments to enhance patient outcomes and oral health.

The review aims to comprehensively examine fabrication technologies for post and core restorations, with a specific focus on customized cast post and cores. Building upon the rationale outlined, the specific objectives of the review are as follows:

- To evaluate the effectiveness of custom-cast post and core restorations in addressing severe tooth damage resulting from conditions such as caries or bruxism;
- To assess the adaptability of custom-cast post and core configurations in cases involving elliptical or flared canals, compared to standard prefabricated posts;
- To investigate the resistance of custom-cast post and core restorations to torsional stresses, highlighting their potential advantages over prefabricated alternatives;
- To explore the role of customized post and core restorations as stabilizing structures between the crown and root, particularly in single-rooted and premolar teeth;
- To examine the ability of tailored post and core restorations to withstand rotational stresses in multi-rooted teeth with significant loss of tooth structure.

By delineating these specific objectives, this review aims to provide a focused analysis of the fabrication technologies for custom-cast post and core restorations and their clinical implications in addressing various dental challenges.

## 2. Search Strategy

**Figure 1:**
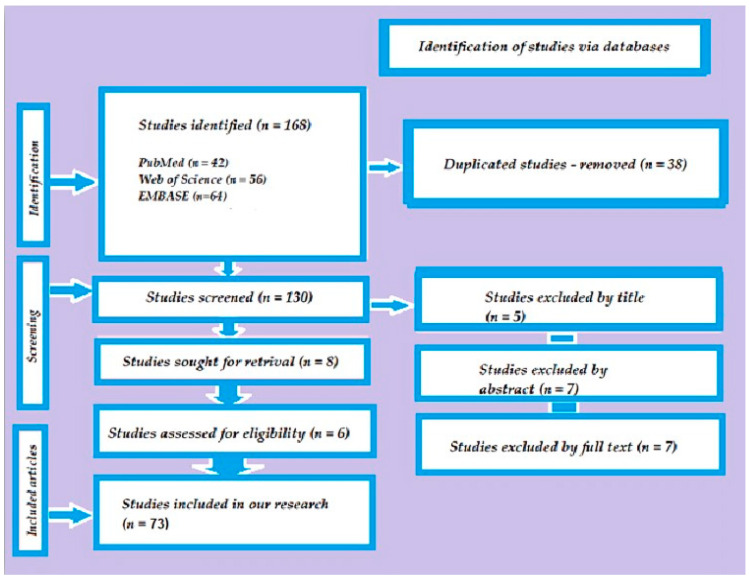
The search strategy of the conducted study.

Using a systematic search strategy, we defined inclusion and exclusion criteria and conducted a thorough search for relevant studies (Figure 1). 

The process involved multiple stages, including

- Database search: studies published between 1993 and 2023 were retrieved from PubMed, Web of Science, and Embase databases. The search criteria included terms such as “Post and core restorations” [Mesh], “Indirect restoration”, “CAD/CAM fabrication”, “Digital dentistry”, and “Post and core” AND “CAD/CAM” [Mesh].
- Selection criteria: out of a total of 168 articles initially screened, 73 met the following specified criteria and were considered for inclusion in the review:
  − Written in English;
  − Published between 1993 and 2023;
  − Addressing post and core restoration techniques, clinical studies, and in vitro studies;
  − Reporting on different fabrication techniques, clinical performance, or quality assessments with conventional and CAD/CAM post and core restorations.
- Exclusion criteria: the 95 articles failing to meet the inclusion criteria or duplicating previously included data were excluded from the review.

During the selection process, the number of articles selected and filtered at each stage was carefully documented. The period between 1 January 1993 and 31 May 2023 was chosen due to the increased availability of clinical and in vitro studies. This rigorous methodology ensured the inclusion of relevant and high-quality literature for the review. Table 1 displays the articles that were included in the review and were deemed highly pertinent to the subject matter at hand.

This narrative review’s search technique included three stages: examining titles, abstracts, and the final selection of publications for full-text analysis. Three reviewers individually sorted articles from databases, and any variations in selection were discussed until a consensus was established. Articles that did not fulfill the predefined inclusion criteria were excluded with the consent of the reviewers. The same reviewers independently reviewed the abstracts of the papers chosen in the second stage, and the articles chosen for the final analysis were received in full text. The whole text of the obtained 73 articles was evaluated in the third and final stage.

## 3. Material Choices for Fabricating Post and Core Restorations

The selection of materials for crafting post and core restorations encompasses a broad classification into metallic and non-metallic options [20]. When it comes to custom-cast post and cores, these are typically fashioned from gold alloys like type III and IV, silver-palladium alloys, or base metal alloys [40,41]. Among these choices, cast gold post and cores stand out as superior due to their remarkable success rates, advantageous mechanical properties, and straightforward manufacturing process [42]. On the other hand, base metal alloys offer a cost-effective alternative, although their stiffness in comparison to dentin can elevate stress levels within the tooth structure [43,44]. Additionally, there are concerns regarding the potential release of harmful chemicals due to the breakdown of base metal alloys [45,46]. Retrospectively, our analysis reveals a success rate ranging from 89% to 98.5% for cast post and cores [47].

Novel materials have emerged by blending ceramics with composite resin, resulting in a hybrid material that boasts the mechanical properties and color stability of ceramics alongside the elasticity and robustness of resin composites [17]. Examples include Enamic, composed of 75% feldspathic porcelain and 25% composite resin, and Lava Ultimate, which incorporates composite resin and 80% nano-ceramic particles [28]. Emerging materials like polyetheretherketone (PEEK) have been explored, owing to their low modulus of elasticity that reduces stress concentration and root fracture risk [9].

The increasing emphasis on achieving aesthetic excellence has sparked the development of ceramic post and core solutions, presenting an alternative to traditional cast options [48]. Notably, castable glass ceramics and glass-penetrated ceramics have garnered attention in this regard [49]. Furthermore, zirconia posts have gained prominence, particularly for teeth with considerable coronal structure loss [50]. Zirconia posts, first introduced in 1995, have demonstrated their capacity to elevate aesthetics and translucency, closely mimicking the appearance of natural teeth [45]. These posts have also exhibited remarkable fracture resistance, surpassing the capabilities of cast metal and glass fiber posts combined with composite resin cores [51]. However, it is crucial to acknowledge that zirconia’s high modulus of elasticity can potentially place amplified stress on root dentin, thereby increasing the risk of root fractures [52]. Moreover, ensuring a secure attachment to acid-resistant zirconia and facilitating the retrieval of zirconia posts in case of treatment failure pose considerable challenges [53].

Multiple research investigations have highlighted a strong association between the effectiveness of restored teeth and the factors encompassing the design, method, and material employed in custom post and core fabrication [5,6,54,55]. In a recent study by Liu et al., findings indicated that CAD-CAM milled post and cores crafted from a cobalt–chromium alloy could serve as a viable substitute for the conventional casting approach in metal post and core fabrication [19]. Nevertheless, it is noteworthy that the retention capabilities of CAD/CAM milled or printed post and cores remained unexamined and untested in the study.

## 4. Comparison between CAD/CAM and Traditional Methods for Post and Core Fabrication

Traditional methods for post and core restorations have been integral to dentistry for an extended period, offering diverse strategies. In the direct approach, the post and core are shaped directly in the patient’s mouth, often utilizing materials such as composite resin or amalgam [8]. While this method is straightforward and cost-effective, it may lack optimal durability. Conversely, the indirect method involves crafting the post and core outside the mouth, typically in a dental laboratory [50]. This begins with an impression of the tooth and its surroundings, facilitating the creation of a personalized restoration using materials like metal alloys or ceramics [17]. Though providing precise fitting, this technique requires additional appointments.

Another technique, the custom-cast post and core, involves tailoring a bespoke post and core using materials like gold or titanium. After preparing the root canal, a precise impression is taken, and a custom post is cast to fit perfectly [43]. Despite offering excellent adaptation, this method may entail multiple visits and laboratory work. Prefabricated post and core systems provide standardized components made of materials like stainless steel or composite resin, offering convenience but potentially compromising fit and strength compared to custom-made options [24].

The choice of technique relies on factors such as the tooth’s condition, the patient’s oral health, and the clinician’s proficiency [11]. Each approach presents its benefits and drawbacks, underscoring the importance of selecting the most appropriate method for each unique case.

The integration of computer-aided design and computer-aided manufacturing (CAD/CAM) technology has sparked a revolutionary transformation across the landscape of dental restoration, reshaping the very foundations of how restorative procedures are approached [54]. Within this digital embrace, a plethora of benefits unfolds, each contributing to the elevation of dental craftsmanship [42]. Precision is elevated to new heights, as CAD/CAM technology operates with meticulous accuracy, leaving minimal room for errors. Consistency is assured through standardized production protocols, ensuring that each restoration adheres to the same exacting standards [18]. The efficiency of the restoration creation process receives a substantial boost, making the journey from digital design to tangible restoration swifter than ever before. Quality control becomes an empowered endeavor, with digital oversight enhancing the assessment of every intricate detail [6]. Perhaps most notably, the capacity to materialize complex structures gains unprecedented traction, enabling the creation of dental restorations that were once confined to the realm of imagination [55].

In its essence, CAD/CAM technology encompasses two fundamental approaches: additive and subtractive manufacturing. The additive pathway, a marvel of modern engineering, brings structures to life by building layer upon layer, each precise deposition contributing to the emergence of intricate forms [3]. In contrast, the subtractive methodology revolves around the art of material removal, sculpting the desired shape by carefully chiseling away. This dichotomy serves as a testament to the versatility that lies at the heart of CAD/CAM technology, offering practitioners a choice in their pursuit of precision [56].

Within the realm of subtractive manufacturing, mechanical robustness is the hallmark of the restorations brought to life [1]. However, this prowess is tempered by the price of material wastage, a reality where a staggering 90% of prefabricated block material often ends up discarded, an unavoidable consequence of the subtractive process [57]. On the flip side, additive manufacturing emerges as the beacon of efficiency, celebrated for its ability to craft intricate structures with an eye for precision while minimizing material wastage. This feature, coupled with the rise of CAD/CAM technology, has prompted a thorough exploration of its capabilities in the realm of custom post and core production [58]. Researchers like Awad and Marghalani blazed a trail in 2007, igniting a journey of discovery that continues through subsequent studies [59,60]. This expedition into the potential of CAD/CAM technology for crafting custom post and cores unfolds through a series of in vitro experiments and case reports, each offering insights into the burgeoning possibilities of this technological marvel [29,30]. As the path forward unfurls, the fusion of dental expertise and digital precision promises to redefine the boundaries of dental restoration, carving a future where innovation and tradition harmoniously coexist [61].

### 4.1. Semi-Digital Indirect Approach

The semi-digital method of post and core fabrication serves as a link between traditional craftsmanship and advanced technology [62]. It begins with a digital scan of either a wax or resin pattern, or an impression of the post space, laying the groundwork for precise fabrication. Once this digital blueprint is established, subsequent steps blend precision and innovation [31,63]. Using milling or additive manufacturing techniques, the cast post and core restoration take form, representing a fusion of digital accuracy and tangible craftsmanship [32,64].

This approach paved the way for the initial exploration into CAD/CAM fabrication of zirconia post and cores, where a digital scan of an acrylic resin pattern marked a significant breakthrough [65,66]. Another aspect involves creating a resin pattern that captures the intricate details of the post area. This pattern undergoes digitization, machining, and sintering, resulting in a unique ceramic post and core that embodies both digital precision and artisanal finesse [33,34]. The exploration continues with variations such as patterns generated from auto-polymerizing resin, followed by scanning and intricate machining steps [67,68].

A divergent iteration in this creative continuum takes shape through the capture of an impression of the cast. A digitized wax pattern, serving as a digital surrogate for the tangible world, takes center stage during the scanning process [65,69]. In the spirit of constant advancement, a recent breakthrough has materialized in the form of scanning a polyvinyl siloxane impression, a precursor to milling a nanoparticle-filled resin block. This progressive technique optimizes chairside efficiency, outshining traditional methods anchored in acrylic resin patterns [54].

Not to be overshadowed, the indirect route of CAD/CAM post and core fabrication embarks on a different yet equally impactful journey. It begins with the creation of an impression of the post space, a critical step that captures the blueprint of the tooth’s intricate structure [70]. This impression transforms into a cast made from scannable stone, a tangible precursor to the digital realm. Guided by this stone cast, the design and milling of the post and core commence, bringing together the physical and digital realms in a harmonious union [33].

### 4.2. Digital Direct Approach

**Figure 2:**
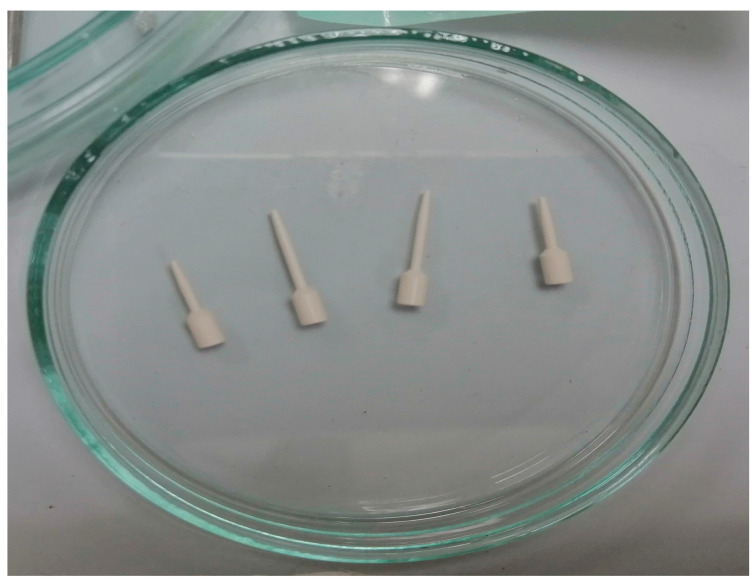
The prepared scan posts before the digital impression (origin of the Figure: author’s own clinical case, no copyright issue) (3Shape patented Scan Posts™, 3Shape, Copenhagen, Denmark).

The entirely digital or direct technique revolves around an optical impression of the post space, achieved through either a digital scan of compatible scan posts coupled with canal-preparing drills, or an intraoral direct scan of the root canal space [71] (Figure 2).

3Shape has introduced specialized Scan Posts™ to streamline the precise recording of post and core restorations’ positions and depths. These Scan Posts™ are versatile, suitable for both intraoral usage in clinics and model scanning in laboratories. They are autoclavable and are available in various shapes and sizes to accommodate drill systems from major suppliers. A patent is pending for this innovation. For input flexibility, the system accepts scans from both TRIOS^®^ intraoral scanners and dental lab scanners [46].

Dentists utilizing 3Shape TRIOS^®^ can initiate post and core cases directly in the clinic by capturing and transmitting reliable input to the lab for direct design. A unique dual-scan process using 3Shape Scan Posts™ ensures accurate capture of root canal depths and positions. When gypsum models are used as the input, lab technicians simply insert Scan Posts™ into the model before scanning [21].

In the laboratory, technicians align the captured Scan Posts™ and allow the software to automatically calculate positions and depths. By initially designing the anatomical layer and utilizing dedicated post and core modeling tools, technicians can craft optimally shaped and functional designs tailored to the clinical case, ready for manufacturing through wax print and cast, milling, or laser sintering [9].

The use of direct digital technology substantially minimizes the clinical timeframe required for crafting chromium–cobalt alloy restorations. This approach effectively eliminates inaccuracies stemming from volumetric alterations in impression materials and plaster used for models, as well as volume fluctuations in wax and composite models [21]. The comprehensive digital method not only saves chairside time but also streamlines laboratory processes by avoiding inaccuracies associated with impression materials and pattern substances like resin and wax [35].

However, despite its advantages, 3D printing does present some challenges. Issues such as polymerization, shrinkage, high resin, and machinery costs in comparison to conventional fabrication methods as well as the potential for surface roughness due to layered resin deposition leading to toxic waste production are still areas of concern [20].

SLA (stereolithography) and DLP (digital light processing) technologies are commonly utilized for printing resins and, on occasion, zirconia in dental applications. However, when dealing with alloys, dental technicians often turn to techniques like SLM (selective laser melting), SLS (selective laser sintering), and other powder bed fusion methods [43]. These techniques are preferred for their ability to precisely fabricate intricate structures from metal powders, ensuring both strength and accuracy in the final product. Moreover, extrusion technology finds extensive use in processing materials such as PEEK (polyetheretherketone), prized for its excellent biocompatibility and mechanical properties, making it suitable for various dental applications, including implant frameworks and temporary prosthetics [14]. 

Additionally, LCM (lithography-based ceramic manufacturing) emerges as a crucial method for producing lithium disilicate ceramics which are, renowned for their aesthetic appeal and robust mechanical properties, making them a preferred choice for dental restorations requiring both beauty and strength [3]. These advanced manufacturing techniques collectively offer dental professionals a diverse array of materials and processes to meet the demanding requirements of modern dentistry [28].

Various materials have been employed for crafting CAD/CAM post and cores [72]. Older studies commonly milled zirconia blocks to create these restorations, whereas newer reports have leaned towards glass fiber-reinforced composites [22,45]. Resin-based post and cores offer a compelling alternative by combining the benefits of traditional custom-made posts and prefabricated fiber posts [12]. Their modulus of elasticity closely resembles that of dentin, potentially leading to more repairable fractures compared to zirconia. Additionally, resin-based materials do not require sintering, allowing for precise fabrication while also reducing production time and cost [33].

A retrospective study found that cast post and cores for single crown restorations had a success rate of 89% to 98.5% after at least seven years of installation. These cast posts and cores can be used to prepare numerous abutments and in patients with significant tooth wear [46]. However, because of their higher modulus of elasticity than dentin, custom-made posts and cores increase the risk of root fracture. They may also cause discoloration in the thin gingival and bone tissue, resulting in a less attractive appearance [47]. Furthermore, if the crown thickness is less than 1.6 mm, the color of the core may influence the outcomes of the translucent ceramic crown [48].

Moustapha et al. conducted a study to determine which digitalization approach provided milled post and cores with the best adaptability [67]. Direct or entirely digital processing using an intraoral scanner, according to reports, results in better adaptability than indirect digitalization of patterns or impressions [36]. Although the direct digitalization technique has an advantage over the indirect technique, recording the limited root canal space during post and core scanning can be problematic at times [37]. 

As the field of dental restoration continues to evolve, the selection of suitable materials for post and core fabrication plays a pivotal role in achieving both functional and aesthetic goals [42]. The balance between mechanical properties, biocompatibility, and long-term stability remains a critical consideration, driving the exploration of innovative materials and manufacturing techniques that can ultimately enhance the longevity and success of these essential dental restorations [14].

### 4.3. Advantages and Disadvantages of Semi-Digital Indirect Approach

#### 4.3.1. Advantages

- Precision and innovation: combines digital accuracy with tangible craftsmanship, resulting in precise restorations [64].
- Versatility: offers various techniques for creating custom post and cores, including scanning resin patterns and impressions [65,67].
- Efficiency: optimizes chairside efficiency, particularly with recent breakthroughs in scanning techniques [54].

#### 4.3.2. Disadvantages

- Complexity of indirect route: involves multiple steps, including creating stone casts and digitizing wax patterns, which can increase complexity [33].
- Limitations in adaptability: limited adaptability compared to direct digital techniques, particularly when scanning the root canal space [37].

### 4.4. Advantages and Disadvantages of Digital Direct Approach

#### 4.4.1. Advantages

- Time efficiency: minimizes clinical time by eliminating inaccuracies associated with traditional impression materials [9].
- Streamlined workflow: reduces inaccuracies in laboratory processes by avoiding the use of impression materials and pattern substances [35].
- Material efficiency in additive manufacturing: additive manufacturing offers material efficiency, reducing waste and costs [14].

#### 4.4.2. Disadvantages

- Challenges in 3D printing: challenges such as polymerization, shrinkage, and high resin costs persist in 3D printing [20].
- Surface roughness in 3D printing: layered resin deposition may result in surface roughness, leading to concerns about toxic waste production [20].
- Limitations in material options: limited material options compared to traditional fabrication methods, with certain materials having durability and fracture resistance concerns [72].
- Potential for root fracture: custom-made posts and cores may increase the risk of root fracture due to their higher modulus of elasticity compared to dentin [47].

As advancements in dental restoration technology continue, selecting appropriate materials and techniques remains crucial for achieving functional and aesthetic success. Balancing factors such as mechanical properties, biocompatibility, and long-term stability drive ongoing exploration into innovative materials and manufacturing techniques to enhance the longevity and effectiveness of post and core restorations [14]. Table 2 highlights the key differences between the semi-digital indirect approach and the digital direct approach in terms of their precision, versatility, workflow efficiency, material efficiency, and adaptability.

## 5. Discussion

The purpose of this study was to undertake a narrative review concerning inquiries into post and core restorations, encompassing their composition, fabrication methodologies, and clinical outcomes.

Numerous reports underscore the capabilities of the CEREC system’s intraoral camera, which demonstrates an impressive capacity to scan post space lengths reaching up to 10 mm [31,32,62,63,64,65]. Consequently, research endeavors often adopt a standardized approach, utilizing a 9 mm post space preparation length for scanning purposes [70]. However, it is noteworthy that when confronted with post gaps surpassing the 10 mm threshold, the prudent course of action leans toward the adoption of an indirect technique for constructing CAD/CAM post and cores. 

The evolution of digital dentistry has aimed to enhance workflow precision and expedite the production process [73]. Initially, the application of CAD-CAM technology for customized posts primarily revolved around scanning plaster models derived from traditional impressions [32]. Alternatively, some researchers proposed an alternative digital approach, wherein a conventional silicone impression is scanned for milling a personalized CAD-CAM post and core [25]. Multiple investigations indicate that both traditional impressions and stone replicas can be successfully digitized with a high degree of reliability [52]. The direct acquisition method was deemed more efficient, accurate, and less invasive compared to indirect techniques [5]. However, achieving precise intraoral scans necessitates the operator’s expertise, experience, and knowledge, and the final outcomes could be influenced by patient-specific variables like intraoral moisture, tongue movement, and saliva flow [67].

An insightful in vitro exploration ventured into the mechanics of CAD/CAM post and cores crafted through direct scanning of the post space, polyether impression scanning, or plaster model scanning. This analytical voyage encompassed measurements of post-retention, cement layer thickness, and nano leakage [66]. The findings unveiled a hierarchy in post retention favoring direct scanning posts and cores, while cement thickness and nano leakage remained generally consistent across the different approaches [73].

Delving into the mechanical realm, one in vitro investigation set its sights on evaluating the push-out bond strength and fracture resistance of various materials deployed in CAD/CAM post and cores. The roster of materials included hybrid ceramic (Enamic), nano-ceramic composite resin (Lava Ultimate), and an experimental glass fiber-reinforced epoxy resin [72,73]. Impressively, the bond strength across the tested materials appeared to be comparable. However, a distinctive pattern emerged in fracture resistance, with the nano-ceramic composite resin reigning supreme, while the hybrid ceramic and glass fiber-reinforced resin showcased parallel values. This dynamic was attributed to the nano-ceramic material’s modulus of elasticity, which closely resembled that of dentin [38]. Moreover, the use of a resin cement seal contributed to more effective biomechanical force distribution, thereby elevating fracture resistance. Intriguingly, all materials demonstrated fracture resistance exceeding the typical adult occlusal force exerted during function, which commonly ranges from 70 N to 150 N [72].

The investigative landscape tilts toward the realm of indirect manufacturing for CAD/CAM post and cores, as highlighted by the majority of documented studies [37,38,39]. While the allure of directly scanning the root canal area lies in its expediency and straightforwardness, the need for indirect methods becomes evident when dealing with teeth boasting extended or diminutive root canal spaces [32,33,64,65,66].

Recent in vitro examinations have ventured into comparisons between milled and 3D-printed Co-Cr alloys against their cast post and core counterparts [29,30,61]. However, the clinical application of CAD/CAM-manufactured Co-Cr alloy remains noticeably absent from the discourse, a void potentially linked to the inherent aesthetic challenges posed by metal alloys, especially when contrasted with the array of alternatives boasting superior aesthetic qualities [73].

Utilizing computer-aided design and computer-aided manufacturing (CAD-CAM) technology, fabricated posts offer a harmonious amalgamation of the benefits of conventional custom posts and prefabricated fiber posts [45]. The incorporation of CAD-CAM-fabricated post and core restorations has been proposed, with specific attention to CAD-CAM fabricated zirconia post and cores garnering research focus [30,73]. Nonetheless, the challenge of retrieving fractured zirconia posts, leading to irreversible tooth failure, adds complexity to their application [38].

Recent advancements have witnessed the integration of resin-based materials into the realm of computer-aided design and computer-aided manufacturing (CAD-CAM) techniques for restoring endodontically treated teeth [10]. The crucial role of restoration adaptation in achieving clinical success has been underscored [24]. CAD-CAM post and core restorations, renowned for their precision, facilitate minimal composite resin cement application, potentially enhancing adhesion to dentinal walls [57]. The meticulous fitting of CAD-CAM-designed posts within the canal not only boasts high esthetic appeal but also accommodates cases with limited coronal remnants and supports extensive prostheses. In comparison to traditional methods, the standout advantage of CAD-CAM techniques lies in the swiftness of preparing restorations, though comprehensive clinical studies are indispensable in assessing their durability over prolonged periods [39].

Acknowledging the boundaries of this study, it is essential to note that one of the key limitations is the challenge of discerning the distinct attributes of various materials. The prevailing dataset leans heavily on clinical reports, predominantly favoring milled zirconia and glass-fiber-reinforced composites as the subjects of analysis. Therefore, a call to action resonates within the scientific community, urging for a more comprehensive exploration of diverse materials and their mechanical characteristics, as the quest to uncover their full potential persists.

These are the most significant key findings from the current review:

- Capabilities of CAD/CAM systems: The CEREC system’s intraoral camera demonstrates impressive scanning abilities for post space lengths up to 10 mm [31,32,62,63,64,65]. A standardized approach utilizing a 9 mm post space preparation length is often adopted for scanning purposes [70].
- Evolution of digital dentistry: Digital dentistry aims to enhance workflow precision and expedite the production process [73]. Initially, CAD-CAM technology for customized posts primarily involved scanning plaster models derived from traditional impressions [32]. Both traditional impressions and stone replicas can be successfully digitized with a high degree of reliability [52].
- Mechanics of CAD/CAM post and cores: In vitro exploration of the mechanics of CAD/CAM post and cores crafted through direct scanning or plaster model scanning reveals a hierarchy in post retention favoring direct scanning posts and cores [66]. The bond strength across tested materials is comparable, but fracture resistance varies, with nano-ceramic composite resin exhibiting the highest resistance [72,73].
- Preference for indirect manufacturing: The majority of documented studies lean towards indirect manufacturing for CAD/CAM post and cores [37,38,39]. While direct scanning of the root canal area is expedient, indirect methods are necessary for teeth with extended or diminutive root canal spaces [32,33,64,65,66].
- Advancements in CAD-CAM technology: CAD-CAM fabricated post and core restorations, particularly zirconia post and cores, have attracted the attention of researchers [30,73]. However, challenges such as retrieving fractured zirconia posts add complexity to their application [38].
- Integration of resin-based materials: Recent advancements integrate resin-based materials into CAD-CAM techniques for restoring endodontically treated teeth [10]. CAD-CAM techniques offer precision and facilitate minimal composite resin cement application, potentially enhancing adhesion to dentinal walls [57].

Key limitations also include the complexities associated with accurately distinguishing the unique characteristics of different materials. Moreover, the reliance on clinical reports predominantly favoring specific materials may inadvertently introduce bias into the analysis. Consequently, the generalizability of the findings might be constrained, hindering their applicability to a broader spectrum of clinical scenarios. Furthermore, the limited scope of available data could impede comprehensive understanding of the diverse factors influencing the efficacy and performance of post and core restorations. Hence, there is a pressing need for further research encompassing a wider range of materials and methodological approaches to ensure more robust and representative findings in this critical area of dental restoration.

Future research should aim to address the limitations identified in the reviewed studies by conducting comprehensive explorations of diverse materials and their mechanical characteristics. This could involve rigorous experimental designs and a broader scope of investigation to provide more robust evidence for clinical decision making. Additionally, longitudinal studies are needed to assess the long-term durability and clinical performance of CAD/CAM post and core restorations.

## 6. Conclusions

The increasing use of CAD/CAM technology in dentistry has extended beyond traditional applications to include post and core restorations, offering a promising alternative to conventional methods. Despite the notable benefits such as enhanced mechanical properties and pleasing aesthetics, evidence from in vivo investigations remains limited. Thus, there is a critical need for comprehensive long-term studies to validate the insights gleaned from existing clinical reports. Clinical recommendations include integrating CAD/CAM-fabricated post and cores into restorative practices, conducting thorough patient assessments, ongoing professional development in CAD/CAM technology, active participation in research endeavors, and fostering collaboration between dental professionals and researchers to advance knowledge and improve patient outcomes.

## Figures and Tables

**Table 1:** Most pertinent articles included in the study.

Author’s Name	Article Type	Type of Impression Technique	Method of Fabrication	Applied Materials	Year of Publication
Hendi at al. [21]	In vitro study	Direct scan	3D Printing	Zirconia	2019
Kanduti et al. [29]	In vitro study	Direct scan	3D Printing	Hybrid ceramic	2021
Marghalani et al. [30]	In vitro study	Direct scan	3D Printing	GFR composite	2012
Sipahi et al. [31]	Clinical case	Impression was taken and scanned	Milling	Zirconia	2011
Liu et al. [32]	In vitro study	Pattern resin made and scanned	Milling	Hybrid ceramic	2010
Lee et al. [33]	Clinical case	Direct scan	Milling	Nano ceramic	2014
Libonati et al. [34]	In vitro study	Pattern resin made and scanned	Milling	Nano-ceramic composite resin	2020
Spina et al. [35]	Clinical case	Pattern resin made and scanned	Milling	GFR composite	2017
Chen at al. [36]	In vitro study	Direct scan	Milling	Base metal Co-Cr alloy	2015
Passos et al. [37]	In vitro study	Pattern resin made and scanned	Milling	Hybrid ceramic	2017
Eid et al. [38]	In vitro study	Direct scan	Milling	Hybrid ceramic	2019
Eid et al. [39]	In vitro study	Direct scan	3D Printing	Zirconia	2021

**Table 2:** Comparison between semi-digital and digital direct methods.

Aspect	Semi-Digital Indirect Approach	Digital Direct Approach
Precision and Accuracy	Combines digital accuracy with tangible craftsmanship	Relies solely on digital impressions and software design
Versatility	Offers various techniques for creating custom post and cores	Limited to digital impressions and software design
Workflow Efficiency	Requires multiple steps including stone casts and digitizing wax patterns	Minimizes clinical and laboratory time by avoiding traditional impression materials
Material Efficiency	Utilizes milling or additive manufacturing techniques	Primarily relies on additive manufacturing methods
Adaptability	May have limitations in adaptability compared to direct digital techniques	May provide superior adaptability, especially when scanning the root canal space

## Data Availability

Data are contained within the article.
